# Chronic consumption of a low calorie, high polyphenol cranberry beverage attenuates inflammation and improves glucoregulation and HDL cholesterol in healthy overweight humans: a randomized controlled trial

**DOI:** 10.1007/s00394-018-1643-z

**Published:** 2018-02-23

**Authors:** Boon Chew, Bridget Mathison, Lindsey Kimble, Diane McKay, Kerrie Kaspar, Christina Khoo, C.-Y. Oliver Chen, Jeffrey Blumberg

**Affiliations:** 1 School of Food Science, Washington State University, Pullman, WA USA; 2 Antioxidants Research Laboratory, Jean Mayer USDA Human Nutrition Research Center on Aging, Tufts University, Boston, MA USA; 3 Ocean Spray Cranberries Inc., Middleboro, MA USA; 4 Nutrition & Food Science, Texas A&M University, College Station, TX USA

**Keywords:** *Vaccinium macrocarpon*, Inflammation, Oxidative stress, Cardiovascular disease, Blood glucose

## Abstract

**Purpose:**

We studied the health benefits of low calorie cranberry beverage consumption on glucoregulation, oxidative damage, inflammation, and lipid metabolism in overweight but otherwise healthy humans.

**Methods:**

78 overweight or obese men and women (30–70 years; BMI 27–35 kg/m^2^) with abdominal adiposity (waist: hip > 0.8 for women and > 0.9 for men; waist: height ≥ 0.5) consumed 450 mL placebo or low calorie, high polyphenol cranberry extract beverage (CEB) daily for 8 week in a randomized, double-blind, placebo-controlled, parallel design trial. Blood and urine samples were collected after overnight fast at baseline and after 8 weeks of daily beverage consumption. Blood and urine samples were also collected during 3 oral glucose tolerance test (OGTT) challenges: (1) pre-intervention without the test beverages, (2) following a single dose of placebo or CEB at baseline (week 0), and (3) following a single dose of placebo or CEB at 8 week.

**Results:**

Compared to placebo, a single CEB dose at baseline lowered endothelin-1 and elevated nitric oxide and the reduced:oxidized glutathione ratio (*P* < 0.05). Interferon-γ was elevated (*P* < 0.05) after a single CEB dose at baseline; however, after 8 week of CEB intervention, fasting C-reactive protein was lower (*P* < 0.05). CEB consumption for 8 week also reduced serum insulin and increased HDL cholesterol compared to placebo (*P* < 0.05).

**Conclusions:**

An acute dose of low calorie, high polyphenol cranberry beverage improved antioxidant status, while 8 week daily consumption reduced cardiovascular disease risk factors by improving glucoregulation, downregulating inflammatory biomarkers, and increasing HDL cholesterol.

**Electronic supplementary material:**

The online version of this article (10.1007/s00394-018-1643-z) contains supplementary material, which is available to authorized users.

## Introduction

Epidemiological studies have shown that the consumption of fruits and vegetables is associated with lower risk of cardiovascular diseases (CVD) and cancer mortality [1–4]. Polyphenols of such plant foods possess an array of bioactivities, e.g., antioxidant, anti-inflammatory, anti-proliferative, that appear to contribute to these observed benefits [5–7]. Cranberries (*Vaccinium macrocarpon*) are a rich source of flavonoids, including proanthocyanidins (PAC), anthocyanins, flavanols, and flavonols, and phenolic acids such as benzoic, hydroxycinnamic, and ellagic acids [8]. While there is a substantial body of evidence regarding the impact of cranberries on urinary tract infections [9], fewer clinical studies have investigated the impact of cranberries on CVD.

To date, studies have explored the effect of cranberry juice on CVD risk factors in healthy subjects [10–15] as well as in patients with type 2 diabetes (T2DM) [16–18], coronary artery disease (CAD) [19], endothelial dysfunction [20], and metabolic syndrome [21]. However, only 2 of these trials have examined people with CVD risk factors other than hypercholesterolemia and T2DM, e.g. overweight/obesity (BMI ≥ 25 kg/m^2^) or central adiposity [22, 23]. In contrast to leaner adults, overweight/obesity increases the susceptibility to CVD because of its association with systemic inflammation, oxidative stress, glucose dysregulation, and dyslipidemia. Particularly, proinflammatory cytokines, such as tumor necrosis factor (TNF)-α and interleukin (IL)-6, produced and secreted from visceral adipose tissue induce insulin resistance, promote oxidative stress, and play a major role in the pathogenesis of endothelial dysfunction and atherosclerosis [24]. Further, impaired glucose tolerance and hyperglycemia have been shown to acutely increase inflammatory cytokine concentrations [25].

Human intervention studies targeting the role of cranberry products in protecting against cardiometabolic risk have produced a mix of positive and null results. Novotny et al. [15] suggested that these inconsistent results may be due to the short length of some of the trials and found that 8 weeks of cranberry juice consumption improved fasting serum triglycerides, C-reactive protein (CRP), diastolic blood pressure, fasting plasma glucose, and insulin resistance in participants with high baseline values. The primary objective of this study was to determine the effects of daily consumption of a low calorie, high-polyphenol cranberry beverage for 8 week on glucoregulation in overweight/obese, but otherwise healthy adults with abdominal adiposity in a double-blind, placebo-controlled, parallel design trial. Other secondary outcomes included indices of oxidative stress, inflammation, and lipid profile. We hypothesized that the consumption of a cranberry extract beverage (CEB) would ameliorate oral glucose tolerance test (OGTT) induced postprandial metabolic dysregulation, which is known to play a role in the development of CVD and T2DM. This approach builds directly upon well-established acute responses in inflammation, antioxidant defenses, and oxidative stress to the OGTT in subjects with both normal and impaired glucose tolerance [25, 26].

## Methods

### Participants and study design

78 non-smoking men and women age 30–70 years with a BMI of 27–35 kg/m^2^, waist:height ratio ≥ 0.5, and waist:hip ratio > 0.8 for women and > 0.9 for men, were recruited between February and November 2012 at two study sites, Washington State University (Pullman, WA) and Tufts University (Boston, MA). The exclusion criteria used to screen for their eligibility included: presence of cardiovascular, endocrine, gastrointestinal, and renal diseases; rheumatologic disorders; active treatment for cancer of any type (except basal cell carcinoma) within the past year; regular use of oral steroids; use of estrogen, with or without progesterone; use of medications known to affect lipid metabolism, blood pressure, or gastrointestinal absorption; systolic blood pressure > 139 mmHg and/or diastolic blood pressure > 89 mmHg; regular use of any dietary supplements within previous 30 days; usual daily ethanol intake of ≥ 2 drinks; cigarette smoking and/or nicotine replacement use; laboratory blood or urine biochemistries (fasting glucose, lipid panel, complete blood count, and urinalysis) outside of normal ranges. The study design was approved by the Institutional Review Boards at Washington State University and Tufts University Health Sciences Campus and Tufts Medical Center. All participants signed a written informed consent agreement before participating. This study was registered with the public registry ClinicalTrials.gov (ID # NCT01527617).

Participants were randomized based on gender, age, and BMI using computer-generated random numbers to receive either one 450 mL serving of placebo or CEB daily for 8 week in a double-blind, parallel trial. The placebo beverage was designed to look, smell, and taste such as the CEB, but did not contain cranberry constituents. Participants received the same test beverage for the duration of the trial. To promote compliance, participants were required to pick up a weekly supply of their assigned beverage and complete a daily compliance calendar to track consumption. Participants were asked to follow a diet low in polyphenol-containing foods for 2-days prior to each sample collection visit, and were provided meals low in polyphenol-containing foods for dinner the night before and lunch during the sample collection visits. Restricted foods included all whole grains, legumes, beans, fruits, berries, vegetables, juices, nuts, seeds, vegetable oils, herbs, spices, tea, coffee, chocolate, and wine. Participants’ usual dietary intake was assessed at the baseline and 8 week visits using the past month Diet History Questionnaire II (Version 2.0. National Institutes of Health, Applied Research Program, National Cancer Institute. 2010) developed by the Risk Factor Monitoring and Methods Branch of the National Cancer Institute [27, 28]. The Diet*Calc analysis Program (Version 1.5.0. National Cancer Institute) was used to interpret the Diet History Questionnaire II data to provide nutrient, food group and diet quality estimates.

**Fig. 1:**
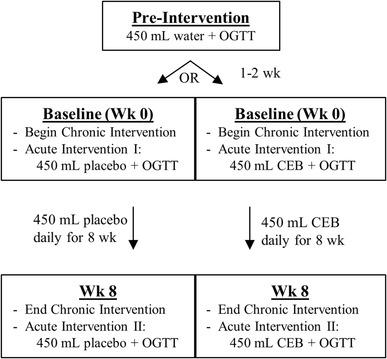
Participants were randomized based on sex and BMI to receive either placebo (*n* = 38) or low calorie, high polyphenol content cranberry extract beverage (CEB; *n* = 40) intervention in a double-blind, placebo-controlled parallel trial. Subjects consumed one 450 mL serving of the assigned study beverage daily during the 8 week intervention period and completed blood and urine sample collection visits at pre-intervention, and following a single dose at baseline (week 0) and week 8 visits

Prior to the baseline visit (1–2 week), a pre-intervention OGTT was conducted to determine glucose and insulin responses without the influence of the test beverages. Following a 2-day low-polyphenol run-in diet and 12-h overnight fast, participants consumed 450 mL water followed by 75 g glucose in 300 mL water, and blood samples were collected via indwelling catheter prior to and at 0.5, 1, 1.5, and 2 h post-consumption for the analysis of serum glucose and insulin (Fig. 1). During the baseline (week 0) and 8 week visits, the OGTT procedure was repeated following a single 450 mL dose of test beverage (in place of the 450 mL water). Additional blood samples were collected at 3, 5, and 8 h post-consumption for the analysis of antioxidant and inflammatory biomarkers. Urine samples were also collected prior to and for 0–8 h post-consumption to quantify polyphenols.

Test beverages were provided by Ocean Spray Cranberries, Inc. (Middleboro, MA). Both beverages were formulated to be low-calorie, contributing 10 kcal per 450 mL serving size (2.5 g carbohydrates). Details of their components are described in Supplemental Table 1.

### Sample preparation

Blood and urine samples were immediately processed and aliquoted following their collection and stored at − 80 °C until analyses. Blood for glucose and insulin analysis was collected into vacutainers, allowed to clot at room temperature for 20 min, then centrifuged (400 ×*g*) to obtain serum. Blood for all other analyses was collected into EDTA vacutainers and centrifuged (400 ×*g*) to obtain plasma and red blood cells (RBC). The 24-h urine samples were collected during the day prior to the study visits and the fasting morning spot urine was collected immediately prior to the administration of the test beverage and OGTT. The 0–8 h urine samples included all urine voided during the 8-h period following the consumption of one test beverage and the OGTT at the baseline (week 0) and 8 week visits. For the analysis of isoprostanes, 8-hydroxydeoxyguanosine (8-OHdG), flavanols, flavonols, phenolic acids, and PAC-A2, 0.025 mol/L BHT (80 µL) was added to urine (5 mL) prior to storage. For the quantification of anthocyanins, urine (10 mL) was stored with 167 µL of 12 mol/L HCl.

### Determination of biochemical biomarkers

Glucose and insulin in serum were quantified using a colorimetric assay (Cayman Chemical Co., Ann Arbor, MI) and a sandwich ELISA (Invitrogen, Camarillo, CA), respectively. The limit of detection (LOD) for insulin was 0.17 µIU/mL. Whole-body insulin sensitivity was assessed using the Matsuda index (ISI-MAT) based on the following formula: 10,000/square root of [fasting glucose (mg/dL) × fasting insulin (µIU/mL) x mean glucose during OGTT × mean insulin during OGTT] [29]. Insulin resistance was assessed using HOMA-IR based on the formula: [fasting insulin (µIU/mL) × fasting glucose (mmol/L)]/22.5 [30].

Total, LDL, and HDL cholesterol (TC, LDL-C, HDL-C) and triglycerides (TG) in plasma were quantified using a clinical chemistry analyzer (Olympus AU400, Center Valley, PA).

Reduced (GSH) and oxidized (GSSG) glutathione in RBC were determined using a commercial colorimetric assay, according to the manufacturer’s instructions (OxisResearch, Portland, OR). The assay was modified to a microtiter plate format (Synergy H1 Hybrid, BioTek, Winooski, VT). The LOD for total GSH was 0.54 µmol/L.

Commercial colorimetric assays were employed to determine the activity of glutathione peroxidase [(GPx) OxisResearch, Portland, OR] and superoxide dismutase [(SOD) Cayman Chemical Co., Ann Arbor, MI] in RBC. The activities were standardized with hemoglobin concentrations determined by colorimetric assay (Arbor Assays, Ann Arbor, MI).

Plasma CRP was quantitated using a sandwich ELISA kit (hs-CRP ELISA Kit, Immuno-Biological Laboratories, Inc., Minneapolis, MN). Plasma IL-6, IL-10, IL-23, interferon-γ (IFN-γ), and TNF-α were analyzed using a multiplex ELISA kit (Quansys, Logan, UT). The LOD was 0.12, 0.15, 0.73, 0.05, and 0.59 pg/mL for IL-6, IL-10, IL-23, IFN-γ, and TNF-α, respectively. Acquired data were analyzed using Quansys Q-View 2.5.2 software.

Plasma oxidized LDL was determined using a commercial ELISA kit (ALPCO Diagnostics, Salem, NH). The LOD was 4.5 ng/mL. The ex vivo resistance of LDL against Cu^2+^-induced oxidation was determined by monitoring the formation of conjugated dienes at 234 nm for 3 h at 37 °C (Shimadzu UV1601 spectrophotometer, Columbia, MD) [31].

F_2α_-isoprostanes in urine were measured using an HPLC and GC/MS method as described by Walter et al. [32]. The final values were adjusted with urinary creatinine to account for differences in urine volume. Urinary 8-OHdG was determined using an API 3200™ LC-MS/MS (Applied Biosystems, Carlsbad, CA) according to Hu et al. [33]. Briefly, 1 mL urine was diluted with formic acid. After the addition of a tracer [9((15)*N*(5)-8-hydroxy-2′-deoxyguanosine], 8-OHdG was extracted using a Waters OASIS 500 mg HLB cartridge and quantified using LC-MS/MS.

Total nitric oxide (NO) in plasma was determined using a commercial kit (Cayman Chemical Co., Ann Arbor, MI). Concentrations were calculated using a nitrate standard curve.

Endothelin-1 (ET-1) was measured in plasma using a sandwich ELISA (Enzo Life Sciences Inc., Farmingdale, NY). The LOD for ET-1 was 0.41 pg/mL.

### Polyphenol bioavailability

PAC-A2 in urine were determined using an LC/MS/MS assay according to McKay et al. [34]. Final PAC-A2 concentrations were adjusted with creatinine to account for urine volume. Anthocyanins were quantified according to Milbury et al. [35, 36] using an API 3200™ LC/MS/MS System. The internal standard employed in the analysis was malvidin-di-glucoside. The concentration of anthocyanins in urine was calculated using standard curves constructed with authentic anthocyanin standards (Extrasynthese, France). The final anthocyanin concentrations were adjusted with creatinine to account for urine volume differences. Urinary flavanols, flavonols, and phenolic acids were quantified by HPLC with electrochemical detection [31, 37]. Concentrations of individual polyphenols were determined using calibration curves constructed with authenticated standards. The internal standard employed in the analysis was 2′,3′,4′-trihydroxyaceophenone. The final analyte concentrations were adjusted with creatinine to account for urine volume.

### Statistical analysis

Results are expressed as means ± SEM. As maximum plasma anthocyanin concentrations have been detected between 1 and 3 h post-consumption [38], we summarize descriptively the curves over time for glucose, insulin, NO and ET-1. Differences in fasting (0 h) values between CEB and placebo were analyzed by ANOVA using the test parameter estimate for the main effect of treatment in our model. Differences following the acute interventions at week 0 and week 8 were assessed separately by ANCOVA with the respective fasting (0 h) values used as a covariate. The chronic effect of CEB consumption at week 8 was assessed by ANCOVA, with the baseline (week 0) values used as a covariate, using the test parameter estimate for the main effect of treatment in our model. Differences were considered significant at *P* < 0.05. The statistical models included study site, age, sex, treatment, time period, BMI, waist:hip ratio, and treatment*time period. All statistical analyses were performed using PROC GLM in SAS version 9.3 (SAS Institute Inc., Cary, NC). The sample size of 40 per group was estimated to detect a 50% reduction in urinary 8-OHdG excretion with a power of 0.70 at the 0.05 level. The sample size calculation was based on levels of urinary 8-OHdG adjusted for creatinine reported by Duthie et al. [11], in which cranberry juice decreased urinary 8-OHdG excretion by 50% from 19.1 ng/mg creatinine with standard deviation of 17.8. The anticipated level of reduction in 8-OHdG is significant as urinary 8-OHdG values in patients with CVD were at least 50% larger than healthy adults [39]. Because there was no available data for the effect of cranberry on glucoregulation at the time the study was designed, 8-OHdG was used as a surrogate for the power calculation.

## Results

### Participants

**Fig. 2:**
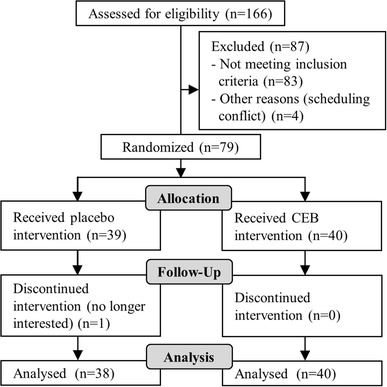
CONSORT diagram for study trial

78 participants completed the study (Fig. 2). There were no significant differences between treatment groups with regard to gender (42% male overall), or age, BMI, waist:hip ratio or waist:height ratio (overall means ± SEM were 43.1 ± 1.1 years, 30.8 ± 0.4 kg/m^2^, 0.91 ± 0.01, 0.62 ± 0.01).

Dietary intake did not differ between the placebo and treatment groups prior to or during the 8 week intervention (Supplemental Table 2). No differences were observed in the level of compliance between groups (98% overall, analyzed by ANOVA), and no participant in either group missed > 2 consecutive beverages during the study.

### Glucoregulation

**Fig. 3:**
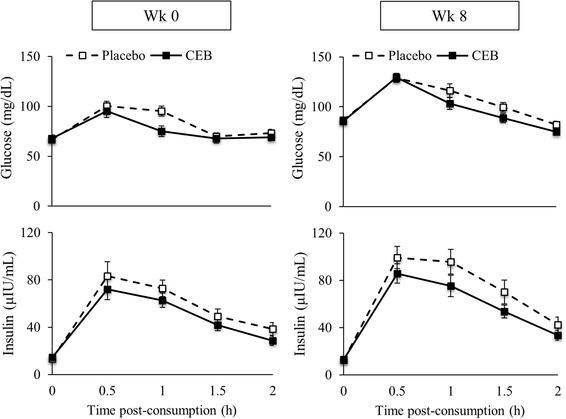
Serum glucose and insulin concentrations (means ± SEM) following a single dose of placebo (*n* = 38) or CEB (*n* = 40) at baseline (week 0) and at 8 week in participants assigned to consume the same test beverage daily for 8 week. Differences following the acute interventions at week 0 and week 8 were assessed separately by ANCOVA with the respective pre-intervention values used as a covariate (*P* < 0.05)

Pre-intervention serum glucose and insulin responses to OGTT were not different between the placebo and treatment groups (data not shown). Following the acute intervention at week 0, the glucose concentration of both groups peaked at 0.5 h post OGTT. The largest observed difference in OGTT curves between treatment groups at week 0 occurred at 1 h for glucose, while insulin values were not different between groups (Fig. 3). Glucose AUC, insulin AUC, and ISI-MAT were not different between groups at week 0 (overall means ± SEM were 160 ± 5 mg/dL/h, 108 ± 6 µIU/mL/h, and 6.5 ± 0.7, respectively). At week 8, the largest observed differences in OGTT curves between treatment groups occurred at 1 h for both glucose and insulin (Fig. 3). There was a significant main effect of treatment (*P* < 0.05) at week 8, with insulin values being lower in the CEB group. Insulin AUC was 21% lower (*P* = 0.06) (119 ± 11 vs. 150 ± 14 µIU/mL/h), while glucose AUC and ISI-MAT were not different between groups at week 8 (overall means ± SEM were 206 ± 5 mg/dL/h and 4.4 ± 0.3, respectively). HOMA-IR values obtained from fasting glucose and insulin values were not different between groups following 8 week of beverage consumption (overall mean ± SEM: 2.77 ± 0.16).

### Lipid metabolism

**Fig. 4:**
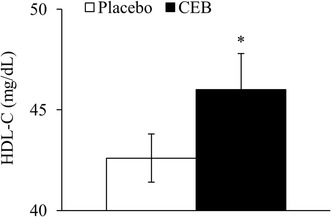
Fasting serum high density lipoprotein cholesterol (HDL-C) concentrations (means ± SEM) following 8 week of placebo (*n* = 38) or CEB (*n* = 40) intervention in participants assigned to consume the same test beverage daily for 8 week. Symbols denote significant difference compared to placebo, analyzed by ANCOVA, with baseline values as a covariate, using the test parameter estimate for the main effect of treatment in our model (*P < 0.05)

Fasting blood lipid profiles at baseline did not differ between groups (data not shown). Fasting HDL-C was 8% higher following 8 week of CEB consumption compared to the placebo group (*P* < 0.05) (Fig. 4). CEB consumption had no effect on fasting TC, LDL-C, or TG (overall means ± SEM were 179.5 ± 3.9, 113.0 ± 5.7, and 112.2 ± 3.2 mg/dL, respectively).

### Oxidative stress and inflammation biomarkers

Fasting (0 h) GSH, GSSG, GSH:GSSG ratio, GPx and SOD values were not different between groups at baseline (week 0). Following a single dose of CEB at week 0 there was a significant main effect of treatment (*P* < 0.05); GSH:GSSG ratio was higher in the CEB group (Table 1). Following a single does at week 8, GSH:GSSG ratio was not different between CEB and placebo. GSH, GSSG, GPx and SOD values were not difference between groups following the acute interventions at week 0 and week 8 (Table 1). After 8 week of beverage consumption, fasting GSH, GSSG, GSH:GSSG ratio, and GPX activity were not different between groups (overall means ± SEM were 1604 ± 53 µmol/L, 85.5 ± 3.8 µmol/L, 20.7 ± 1.9, and 10.0 ± 0.3 mU/mg HgB, respectively). However, fasting SOD activity tended to be 11% higher (*P* = 0.07) in the CEB than placebo group (1.91 ± 0.07 vs. 1.72 ± 0.05 U/mg HgB).

**Table 1:** Biomarkers of antioxidant capacity following a single dose of placebo (*n* = 38) or CEB (*n* = 40) at baseline (week 0) and at 8 week in participants assigned to consume the same test beverage daily for 8 week

	Hours	Week 0				Week 8			
		Placebo	CEB	Effect	*P*	Placebo	CEB	Effect	*P*
GSH, µmol/L	0	1373 ± 72	1373 ± 67			1538 ± 80	1668 ± 71		
	3	1371 ± 66	1382 ± 59	Trt	0.084	1602 ± 80	1605 ± 59	Trt	0.886
	5	1354 ± 74	1382 ± 52	Time	0.076	1587 ± 93	1616 ± 72	Time	0.934
	8	1407 ± 64	1530 ± 47	T × T	0.467	1554 ± 103	1607 ± 67	T × T	0.940
GSSG, µmol/L	0	64.5 ± 6.5	69.3 ± 6.0			88.0 ± 5.1	83.1 ± 5.7		
	3	65.0 ± 6.1	69.4 ± 6.4	Trt	0.832	79.1 ± 5.6	81.8 ± 5.1	Trt	0.604
	5	66.6 ± 5.6	64.8 ± 6.8	Time	0.598	82.7 ± 6.1	80.2 ± 5.6	Time	0.001
	8	60.5 ± 4.9	64.3 ± 7.2	T × T	0.778	65.7 ± 5.8	60.1 ± 5.4	T × T	0.734
GSH:GSSG ratio	0	25.3 ± 2.8	21.7 ± 2.4			17.4 ± 1.5	23.9 ± 3.5		
	3	23.0 ± 1.9	26.9 ± 6.3	Trt	0.016	22.4 ± 2.7	24.2 ± 5.4	Trt	0.790
	5	22.6 ± 2.5	33.8 ± 8.0	Time	0.272	20.4 ± 2.2	20.9 ± 1.7	Time	0.008
	8	25.5 ± 2.6	43.8 ± 10.3	T × T	0.498	32.9 ± 6.4	36.3 ± 7.8	T × T	0.954
GPx, mU/mg HgB	0	10.3 ± 0.6	11.3 ± 0.9			9.7 ± 0.4	10.4 ± 0.5		
	3	10.0 ± 0.7	11.1 ± 0.6	Trt	0.937	10.1 ± 0.6	10.0 ± 0.4	Trt	0.126
	5	10.5 ± 0.6	11.4 ± 0.8	Time	0.760	10.0 ± 0.6	10.0 ± 0.5	Time	0.753
	8	10.8 ± 1.0	10.8 ± 0.7	T × T	0.566	9.9 ± 0.5	9.6 ± 0.5	T × T	0.932
SOD, U/mg HgB	0	1.71 ± 0.07	1.85 ± 0.08			1.72 ± 0.05	1.91 ± 0.07		
	3	1.80 ± 0.07	1.96 ± 0.08	Trt	0.230	1.74 ± 0.06	1.84 ± 0.06	Trt	0.393
	5	1.80 ± 0.08	1.91 ± 0.07	Time	0.363	1.65 ± 0.06	1.83 ± 0.08	Time	0.633
	8	1.73 ± 0.05	1.88 ± 0.08	T × T	0.855	1.70 ± 0.06	1.82 ± 0.07	T × T	0.707

Values are means ± SEM

Differences following the acute interventions at week 0 and week 8 were assessed separately by ANCOVA with the respective fasting (0 h) values used as a covariate (*P* < 0.05)

Fasting (0 h) CRP, IL-6, IL-10, IL-23, TNF-α, and IFN-γ values were not different between groups at baseline (week 0). Following a single dose of CEB at week 0, there was a significant main effect of treatment (*P* < 0.05); IFN-γ values were higher in the CEB group (Table 2). However, a single dose of CEB at week 0 had no effect on the other inflammatory biomarkers, i.e., CRP, IL-6, IL-10, IL-23, and TNF-α (Table 2). A single dose of CEB at week 8 had no effect on any inflammatory biomarkers (Table 2). Fasting CRP was 22% lower (*P* < 0.05) following 8 week of CEB consumption (2.55 ± 0.29 µg/mL) than in the placebo group (3.27 ± 0.51 µg/mL). Fasting concentrations of IL-6, IL-10, IL-23, TNF-α, and IFN-γ were not statistically different between groups following 8 week of beverage consumption (overall means ± SEM were 3.88 ± 0.28, 6.11 ± 0.67, 30.8 ± 2.1, 7.52 ± 0.38, and 0.99 ± 0.10 pg/mL, respectively).

**Table 2:** Plasma inflammatory and oxidative stress biomarkers following a single dose of placebo (*n* = 38) or CEB (*n* = 40) at baseline (week 0) and at 8 week in participants assigned to consume the same test beverage daily for 8 week

	Hours	Week 0				Week 8			
		Placebo	CEB	Effect	*P*	Placebo	CEB	Effect	*P*
CRP, µg/mL	0	2.81 ± 0.28	3.48 ± 0.45			3.27 ± 0.51	2.55 ± 0.29		
	3	2.58 ± 0.32	3.60 ± 0.48	Trt	0.334	3.09 ± 0.45	2.70 ± 0.34	Trt	0.171
	5	2.58 ± 0.29	3.39 ± 0.51	Time	0.680	3.10 ± 0.44	2.63 ± 0.27	Time	0.670
	8	2.74 ± 0.38	3.59 ± 0.56	T × T	0.858	2.82 ± 0.41	2.64 ± 0.33	T × T	0.746
IL-6, pg/mL	0	4.88 ± 0.53	4.64 ± 0.50			4.12 ± 0.44	3.66 ± 0.35		
	3	5.60 ± 0.58	5.21 ± 0.52	Trt	0.750	4.63 ± 0.48	4.27 ± 0.42	Trt	0.531
	5	6.72 ± 0.66	7.02 ± 0.71	Time	0.001	5.12 ± 0.50	5.08 ± 0.46	Time	0.001
	8	7.93 ± 0.71	7.33 ± 0.66	T × T	0.410	6.62 ± 0.71	5.98 ± 0.54	T × T	0.593
IL-10, pg/mL	0	5.60 ± 0.90	5.96 ± 0.94			5.73 ± 0.91	6.46 ± 1.00		
	3	5.46 ± 0.90	5.87 ± 0.99	Trt	0.379	5.41 ± 0.89	6.34 ± 1.05	Trt	0.713
	5	5.45 ± 0.87	6.21 ± 0.97	Time	0.136	6.26 ± 1.08	6.64 ± 1.04	Time	0.221
	8	5.98 ± 0.89	6.38 ± 1.00	T × T	0.727	5.48 ± 0.97	6.57 ± 1.04	T × T	0.550
IL-23, pg/mL	0	27.1 ± 2.2	28.3 ± 2.9			30.1 ± 3.1	31.6 ± 3.1		
	3	26.7 ± 2.3	29.7 ± 3.4	Trt	0.051	29.9 ± 3.0	30.5 ± 3.0	Trt	0.123
	5	27.6 ± 2.5	30.1 ± 3.1	Time	0.617	27.3 ± 2.4	31.1 ± 3.1	Time	0.354
	8	27.5 ± 2.3	31.0 ± 3.3	T × T	0.920	29.2 ± 2.7	33.1 ± 3.1	T × T	0.387
TNF-α, pg/mL	0	7.48 ± 0.36	8.61 ± 0.53			7.16 ± 0.52	7.86 ± 0.56		
	3	7.50 ± 0.40	8.70 ± 0.54	Trt	0.132	6.85 ± 0.48	7.68 ± 0.52	Trt	0.267
	5	7.20 ± 0.43	8.94 ± 0.65	Time	0.667	6.53 ± 0.49	7.01 ± 0.50	Time	0.057
	8	7.62 ± 0.41	9.00 ± 0.63	T × T	0.640	6.81 ± 0.50	7.65 ± 0.56	T × T	0.664
IFN-γ, pg/mL	0	1.01 ± 0.11	1.01 ± 0.16			1.11 ± 0.18	0.88 ± 0.11		
	3	0.89 ± 0.10	0.96 ± 0.15	Trt	0.004	0.97 ± 0.13	0.75 ± 0.07	Trt	0.055
	5	0.88 ± 0.10	0.96 ± 0.16	Time	0.015	0.98 ± 0.13	0.75 ± 0.07	Time	0.454
	8	0.97 ± 0.12	1.07 ± 0.17	T × T	0.892	1.05 ± 0.14	0.79 ± 0.07	T × T	0.922

Values are means ± SEM

Differences following the acute interventions at week 0 and week 8 were assessed separately by ANCOVA with the respective fasting (0 h) values used as a covariate (*P* < 0.05)

Fasting (0 h) circulating oxidized LDL and ex vivo resistance of LDL to Cu^2+^-induced oxidation (expressed as lag time) values at baseline (week 0) were not different between groups (data not shown). A single dose of CEB had no effect on circulating oxidized LDL or ex vivo resistance of LDL to Cu^2+^-induced oxidation (expressed as lag time) at week 0 (overall means ± SEM were 82.6 ± 8.7 ng/mL and 83.8 ± 1.5 min, respectively) and week 8 (overall means ± SEM were 85.4 ± 9.9 ng/mL and 78.8 ± 1.4 min). Chronic consumption of the study beverages for 8 week did not affect fasting concentrations of circulating oxidized LDL and lag time (overall means ± SEM were 89.0 ± 9.9 ng/mL and 78.2 ± 1.4 min, respectively). Similarly, 8-OHdG and isoprostanes in 24-h urine samples collected during the day prior to the week 0 and 8 visits were not different (overall means ± SEM were 4.2 ± 0.2 and 11.4 ± 0.4 ng/mg creatinine, respectively).

**Fig. 5:**
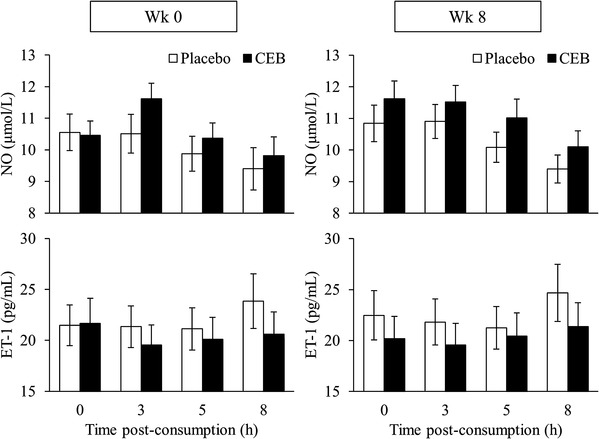
Plasma NO and ET-1 concentrations (means ± SEM) following a single dose of placebo (*n* = 38) or CEB (*n* = 40) at baseline (week 0) and at 8 week in participants assigned to consume the same test beverage daily for 8 week. Differences following the acute interventions at week 0 and week 8 were assessed separately by ANCOVA with the respective fasting (0 h) values used as a covariate (*P* < 0.05)

Fasting (0 h) NO and ET-1 values were not different between groups at baseline (week 0). At week 0, the largest observed differences in NO and ET-1 curves between treatment groups occurred at 3 h (Fig. 5). Following a single dose of CEB at week 0, there were significant main effects of treatment (*P* < 0.05); NO values were higher and ET-1 values were lower in the CEB group (Fig. 5). However, a single dose of CEB at week 8 had no effect on NO or ET-1. Additionally, chronic consumption of the study beverages for 8 week did not affect fasting concentrations of NO and ET-1 (overall means ± SEM were 11.2 ± 0.4 µmol/L and 21.3 ± 1.6 pg/mL, respectively).

### Polyphenol status

Pre-consumption concentrations of PAC-A2 in pooled 24-h urine samples collected during the day prior to the week 0 visit did not differ between the two groups (overall mean 126 ± 14 pg/mg creatinine). Urinary PAC-A2 concentrations were 67% higher (*P* < 0.05) in pooled 24 h urine samples following CEB consumption after 8 week, as compared to placebo (200 ± 30 vs. 120 ± 20 pg/mg creatinine). At week 0 and 8, a single acute dose of CEB increased urinary excretion of cyanidin-3-arabinoside, galactoside, and glucoside and peonidin-3-arabinoside, galactoside, and glucoside by ≥ 29-, 8-, 4-, 30-, 26-, and 3-fold, respectively, in pooled samples collected 0–8 h post-consumption, as compared to fasting (0 h) values (*P* < 0.05) (Table 3). Anthocyanins in fasting morning spot urine were not elevated after the 8 week consumption of CEB compared to baseline (week 0) values. Anthocyanins were not determined in urine collected from the placebo group.

**Table 3:** Concentrations (ng/mg creatinine) of anthocyanins in urine samples collected following a single dose of CEB (*n* = 40) at baseline (week 0) and at 8 week in participants assigned to consume CEB daily for 8 week

	Week 0		Week 8	
	0 h	0–8 h	0 h	0–8 h
Cyanidin-3-arabinoside	0.02 ± 0.01	2.18 ± 0.20*	0.08 ± 0.02	2.39 ± 0.19*
Cyanidin-3-galactoside	0.19 ± 0.06	3.23 ± 0.42*	0.33 ± 0.13	2.88 ± 0.33*
Cyanidin-3-glucoside	0.93 ± 0.40	6.74 ± 1.04*	1.40 ± 0.50	7.13 ± 1.19*
Peonidin-3-arabinoside	0.07 ± 0.04	8.74 ± 0.76*	0.31 ± 0.09	9.56 ± 0.91*
Peonidin-3-galactoside	0.17 ± 0.06	20.9 ± 1.6*	0.91 ± 0.25	24.5 ± 1.9*
Peonidin-3-glucoside	0.27 ± 0.08	1.33 ± 0.16*	0.34 ± 0.06	1.44 ± 0.16*

Values are means ± SEM

Anthocyanins were not determined in urine collected from the placebo group

Symbols denote significant difference within treatment compared to fasting (0 h) values of morning spot urine samples, analyzed at week 0 and week 8 separately by ANOVA using the test parameter estimate for the main effect of time in our model (*P < 0.05)

Fasting (0 h) concentrations of flavanols, flavonols, and phenolic acids were not different between groups at baseline. Following a single dose of CEB at week 0 and 8, concentrations of epicatechin and caffeic, *p*-coumaric, ferulic, sinapic, and 4-hydroxy-3-methoxyphenylacetic acids in pooled urine collected between 0 and 8 h post-consumption were increased compared to the placebo group (*P* < 0.05) (Table 4). The magnitude of the differences ranged from 0.3- to 17.2-fold at week 0 and from 0.6- to 20.6-fold at week 8. At the week 8 visit, a single dose of CEB led to 70 and 80% larger urinary excretion of gentisic and 3,4-dihydroxyphenylacetic acids (*P* < 0.05) (Table 4). The quantified flavanols, flavonols, and phenolic acids in the fasting morning spot urine were not affected by the consumption of CEB for 8 week (Supplemental Table 3). Compared with the values at week 0, CEB consumption for 8 week increased urinary epicatechin and caffeic, *p*-coumaric, ferulic, sinapic, 3,4-dihydroxyphenylacetic, 4-hydroxy-3-methoxyphenylacetic, and gentisic acids by 400, 125, 270, 89, 121, 16, 15, and 51%, respectively, in 24-h pooled urine samples collected prior to week 8 (*P* < 0.05) (Supplemental Table 4). Flavanols, flavonols, and phenolic acids were not measured in 24-h pooled urine samples collected from the placebo group. CEB consumption did not change urinary excretion of 4-hydroxyphenylacetic or 3-hydroxybenzoic acids. Catechin, myricetin, isorhamnetin, and protocatechuic, 4-hydroxybenzoic, and vanillic acids were largely undetected.

**Table 4:** Concentrations (ng/mg creatinine) of flavanols, flavonols, and phenolic acids in pooled urine samples collected up to 8 h following a single dose of placebo (*n* = 38) or CEB (*n* = 40) at baseline (week 0) and at 8 week in participants assigned to consume the same test beverage daily for 8 week

	Week 0		Week 8	
	Placebo	CEB	Placebo	CEB
Epicatechin	125 ± 33	211 ± 35*	158 ± 33	307 ± 34*
Quercetin	546 ± 54	636 ± 56	499 ± 58	684 ± 76
Caffeic acid	53 ± 14	95 ± 19*	35 ± 8	103 ± 21*
p-Coumaric acid	20 ± 9	364 ± 33*	29 ± 12	411 ± 33*
Ferulic acid	419 ± 66	907 ± 93*	402 ± 60	911 ± 73*
Sinapic acid	18 ± 5	286 ± 49*	13 ± 0	281 ± 34*
4-OH-PAA	13,435 ± 1909	12,902 ± 1918	15,247 ± 1619	13,777 ± 1647
3,4-OH-PAA	428 ± 59	722 ± 162	370 ± 49	665 ± 88*
4-OH-3-MeOH-PAA	3498 ± 251	4676 ± 405*	3579 ± 281	5654 ± 486*
3-OH-Benzoic acid	4434 ± 543	5243 ± 626	5611 ± 719	5332 ± 713
Gentisic acid	124 ± 25	173 ± 21	141 ± 28	234 ± 34*

Values are means ± SEM

Symbols denote significant difference compared to placebo, analyzed by ANCOVA, with the respective fasting (0 h) values as a covariate, using the test parameter estimate for the main effect of treatment in our model (*P < 0.05)

## Discussion

### Glucoregulation

Cranberry juice is rich in polyphenols that may diminish postprandial glycemic responses to co-consumed foods and improve glucoregulation after chronic consumption by slowing gastric uptake of glucose or enhancing the distribution of glucose to insulin-sensitive tissues [18]. A recent study of Chinese patients with T2DM reported that 24-week supplementation with 160 mg/day anthocyanins from bilberries and blackcurrants lowered fasting plasma glucose and HOMA-IR [40]. Furthermore, Novotny et al. [15] noted in an 8 week intervention with overweight, older adults that low calorie cranberry juice (240 mL/day) reduced fasting glucose and improved HOMA-IR in individuals with a high baseline value. However, we did not find that CEB consumption for 8 week improved fasting glycemic parameters in healthy overweight adults. Similarly, cranberry juice consumption for 4 week did not affect fasting glucose, insulin, or HOMA-IR in subjects with CAD [19]. Thus, the long-term benefit of cranberry polyphenols on fasting glycemic control may be more evident in people with impaired glucose control. Nevertheless, we found that polyphenols in CEB were beneficial to postprandial glucoregulation measured using OGTT. Interestingly, serum glucose concentrations at all time points during the OGTT were higher in both the placebo and CEB groups at week 8 than at week 0, probably affecting the probability of detecting the glucoregulation effect of CEB at week 8. While the mechanisms remain to be explored, we speculate that CEB polyphenols may enhance the sensitivity of tissues to the effects of insulin as less insulin was required for the excursion of the same amount of ingested glucose.

### Lipid metabolism

Dyslipidemia is a critical risk factor for CVD. In the present study, daily CEB for 8 week increased fasting plasma HDL-C by ~ 3 mg/dL, but did not affect other lipid parameters. Gordon et al. [41] reported that for each 10 mg/L (1 mg/dL) HDL-C increase, CVD risk was reduced by 2–3%, corresponding to a 6–9% risk reduction in this study. These results are in agreement with Ruel et al. [23], who reported that cranberry juice cocktail consumption only increased HDL-C by 8% in men with abdominal adiposity. However, several human trials with a wide range of subject characteristics and study designs show a null effect of cranberry juice on the lipid profile [11, 12, 16, 19–21]. Differences in the study populations, baseline subject characteristics, medication use, the polyphenol composition of the juice, and the duration of intervention may underlie differences seen in lipid profiles following cranberry juice consumption. Although the constituents exerting the lipid lowering effect remain to be elucidated, anthocyanins have been reported to improve HDL-C and LDL-C in dyslipidemic subjects [42, 43] through the inhibition of cholesterol ester transfer protein [42].

### Oxidative stress and inflammation biomarkers

Enhanced oxidative stress and oxidative modification of lipids, proteins, and nucleic acids contribute to the pathogenesis of CVD [12]. The evidence that cranberry consumption increases antioxidant capacity and decreases biomarkers of oxidative stress is equivocal. Cranberry juice has been shown to increase plasma antioxidant capacity and decrease oxidized LDL in healthy men [12] and in women with metabolic syndrome [21]. The reduction in oxidized LDL is beneficial to CVD as the accumulation of oxidized LDL inside macrophages contribute to development and progression of atherosclerosis. However, in people with endothelial dysfunction and CVD risk factors [20], in overweight men [22], and in patients with T2DM taking oral glucose-lowering medications [17], cranberry juice or cranberry extract powder were not shown to increase the resistance of LDL to oxidation. In this study, we found neither a single dose nor 8 week consumption of CEB impacted circulating oxidized LDL and LDL resistance to *ex vivo* Cu^2+^-induced oxidation. Similarly, no effect was noted on urinary isoprostanes and 8-OHdG, systemic biomarkers of lipid peroxidation and DNA oxidation.

Polyphenols and flavonoids regulate the expression of antioxidant enzymes and production of endogenous small molecule antioxidants; e.g., GSH [44]. In the present study, a single dose of CEB increased GSH:GSSG ratio in RBC in a manner consistent with the work of Mathison et al. [14], which showed that a single dose of cranberry juice increased GSH concentrations after 24 h in healthy adults. However, we did not find the same effect after the 8 week CEB consumption. These results are consistent with a temporal effect of polyphenols on elevated GSH concentrations reported by Chen et al. [37]. In addition to GSH:GSSG ratio, we noted that SOD activity but not GPx was greater after chronic CEB consumption. These results are consistent with Duthie et al. [11] and Valentova et al. [45].

Inflammation plays an important role in the development and progression of multiple cardiovascular conditions. The reduction in CRP after chronic CEB consumption suggests an improvement of inflammatory status in these older and overweight/obese adults. CRP is recognized by the American Heart Association and the Center for Disease Control as a predictive factor for cardiovascular disease risk [46]. Guidelines indicate that CRP levels < 3 mg/L are associated with a low risk CVD risk, 3–10 mg/L moderate risk, and > 10 mg/L high risk [47]. Based on these criteria, 8 week of CEB resulted in a low CVD risk classification (2.55 mg/L), whereas placebo CRP levels were categorized as moderate risk (3.27 mg/L). In a recent study, Novotny et al. [15] also showed that cranberry juice reduced CRP concentrations in overweight adults. Both trials are concordant with a NHANES 2005–2008 dietary survey data that indicated CRP levels are lower in cranberry juice consumers compared to non-consumers [48]. Other trials of cranberry juice have revealed no change in inflammatory status [17, 19–21]. The acute CEB dose increased IFN-γ, an inflammatory mediator secreted by Th1 cells which activate monocyte/macrophages, natural killer cells, and cytotoxic T cells in association with host defenses against bacteria, viruses and fungi [49]. This result is in contrast to results from an *in vitro* whole blood experiment in which kaempferol inhibited concanavalin A-simulated IFN-γ production [50]. Nonetheless, confirming the effect of CEB on IFN-γ is warranted along with further exploration of the effect of cranberry polyphenols on the regulation of immunity against microbial infections.

CVD risk factors such as overweight/obesity, dyslipidemia, hypercholesterolemia, and elevated CRP are associated with endothelial dysfunction [51]. Endothelium-derived NO and ET-1 play an important role in the modulation of vascular tone. We found an acute dose of CEB increased NO and decreased ET-1, suggesting a temporal benefit to vasodilation. Dohadwala et al. [19] reported an improvement in vasodilation 2–4 h after a single dose of cranberry juice in an uncontrolled pilot trial, but not following daily consumption (480 mL/day) for 4 week in patients with CAD. Canton et al. [52] and Yung et al. [53] suggested that procyanidin-rich foods and beverages might decrease ET-1 and increase NO via modulation of their syntheses. However, Mathison et al. [14] reported that a single dose of cranberry beverage had no effect on plasma NO in healthy adults and Flammer et al. [20] found no change in endothelial function after consumption of a double-strength cranberry juice (460 mL/day) for 4 months in patients with CVD risk factors and endothelial dysfunction.

### Polyphenol status

Flavonoids and phenolic acids in cranberry have been detected in blood and urine following cranberry consumption [34, 38, 45, 54–56], in addition to PAC-A2 in urine [34]. Peonidin-3-galactoside was the most abundant anthocyanin in the urine, though part of it may be derived via methylation of absorbed cyanidin-galactoside. An acute dose of CEB was found to elevate urinary epicatechin, hydroxycinnaminic acids, and 4-hydroxy-3-methoxyphenylacetic acid with 4-hydroxy-3-methoxyphenylacetic, ferulic, and *p*-coumaric acids being the most abundant. Similarly, McKay et al. [34] found that *p*-coumaric, 4-hydroxy-3-methoxyphenylacetic, 4-hydroxyphenylacetic, and 3,4-dihydroxyphenylacetic acids were abundant in urine following a single dose of cranberry juice in an uncontrolled trial in healthy older adults.

While most studies examining cranberry phenolic bioavailability focus on uptake and excretion following a single dose, chronic CEB consumption in the present study increased gentisic acid, epicatechin, hydroxycinnaminic acids, 4-hydroxy-3-methoxyphenylacetic and 3,4-dihydroxyphenylacetic acids in pooled 24 h urine samples collected during the day prior to the final intervention. While phenolic acids are abundant in cranberry juice, they are also generated in the GI tract via non-specific degradation and microbiota-mediated polyphenol catabolism, such as protocatechuic acid from cyanindin-3-glucoside [57]. However, protocatechuic acid was not detected in the urine of this study, probably due to a lower concentration of anthocyanins in the CEB than the test product used by Czank et al. [57]. Further, following the intake of grape fiber, Saura-Calixto et al. [58] demonstrated hydroxyphenylacetic acids as major metabolites produced via colonic fermentation of non-extractable PAC and bioavailable to plasma. Consistent with this observation, we detected these compounds in urine after acute and chronic CEB consumption. Since cranberry phenolics are absorbed and metabolized at different locations within the GI tract via endogenous detoxification mechanisms and bacterial enzymes, the large inter-individual variation in urinary polyphenol profiles could be a consequence of different rates of metabolism and gut microbiota composition [54, 59]. These differences may partially explain the wide range of bioefficacy found in clinical trials of polyphenols.

In conclusion, consumption of a low calorie, high polyphenol CEB reduced CVD risk factors by improving redox status, vasodilation, glucose homeostasis, and HDL cholesterol in healthy overweight/obese adults. An acute CEB dose improved endogenous antioxidant defenses and vasodilation, while 8 week chronic consumption improved glucoregulation and HDL cholesterol, and down-regulated CRP. Population characteristics, baseline biomarker status, composition of the test products, and duration of the intervention may all be important factors when investigating the health benefits of cranberries.

## Electronic supplementary material

Below is the link to the electronic supplementary material.

Supplementary material 1 (DOCX 32 KB)
