# Supplementary material for: Chronic consumption of a low calorie, high polyphenol cranberry beverage attenuates inflammation and improves glucoregulation and HDL cholesterol in healthy overweight humans: a randomized controlled trial
Source: Eur J Nutr. 2018 Feb 23;58(3):1223–35. doi: 10.1007/s00394-018-1643-z (PMC6499871; doi:10.1007/s00394-018-1643-z)
Supplement: Supplementary file 1 — Supplementary material 1 (DOCX 32 KB) [file 394_2018_1643_MOESM1_ESM.docx]

**Supplemental Table 1** Composition of treatment beverages

|  | Placebo | CEB |
| --- | --- | --- |
| Calories (kcal) | 10 | 10 |
| Carbohydrates (g) | 2.5 | 2.5 |
| Protein (g) | 0 | 0 |
| Fat (g) | 0 | 0 |
| Vitamin C^a^ | ND | ND |
| PAC (OSC-DMAC)^b^ | ND | 145 |
| PAC (BL-DMAC)^c^ | ND | 49.6 |
| Total anthocyanins^d^ | ND | 6.22 |
| Cyanidin-3-arabinoside | ND | 1.52 |
| Cyanidin-3-galactoside | ND | 1.78 |
| Cyanidin-3-glucoside | ND | 0.07 |
| Peonidin-3-arabinoside | ND | 0.80 |
| Peonidin-3-galactoside | ND | 1.77 |
| Peonidin-3-glucoside | ND | 0.22 |
| Total phenolics^e^ | ND | 8.82 |
| Epicatechin | ND | 2.14 |
| Catechin | ND | 0.09 |
| Quercetin | ND | 2.06 |
| Myricetin | ND | 1.25 |
| Isorhamnetin | ND | ND |
| Caffeic acid | ND | 0.25 |
| p-Coumaric acid | ND | 1.26 |
| Ferulic acid | ND | 0.13 |
| Sinapic acid | ND | 0.36 |
| 4-OH-PAA | ND | ND |
| 3,4-OH-PAA | ND | 0.01 |
| 4-OH-3-MeOH-PAA | ND | 0.45 |
| 3-OH-Benzoic acid | ND | 0.07 |
| 4-OH-Benzoic acid | ND | 0.11 |
| Gentisic acid | ND | 0.02 |
| Protocatechuic acid | ND | 0.36 |
| Vanillic acid | ND | 0.27 |

Values are mg per 450 mL serving size unless otherwise indicated

^a^Vitamin C was measured using an iodiometric titration method [1]

^b^PAC concentration was initially quantified using a modified colorimetric method (OSC-DMAC) described by Martin et al [2]

^c^PAC were also analyzed using the colorimetric method (BL-DMAC) described by Prior et al [3] with PAC-A2 as a reference standard

^d^Anthocyanins were analyzed by HPLC (Agilent 1100; Agilent Technologies, Inc, Santa Clara, CA) as described by Martin et al [2]

^e^Concentrations of phenolics were measured by HPLC [4, 5]

**Supplemental Table 2** Usual dietary intake during the prior month collected at baseline (wk 0) and following 8 wk daily consumption in participants assigned to consumed placebo (*n* = 38) or CEB (*n* = 40)

|  | Wk 0 | |  | Wk 8 | |
| --- | --- | --- | --- | --- | --- |
|  | Placebo | CEB |  | Placebo | CEB |
| Energy (kcal) | 2501 ± 212 | 2337 ± 229 |  | 1974 ± 156 | 1799 ± 133 |
| Total fat (g) | 106 ± 11 | 99 ± 10 |  | 83 ± 7 | 74 ± 6 |
| Carbohydrate (g) | 275 ± 23 | 268 ± 28 |  | 222 ± 18 | 206 ± 15 |
| Protein (g) | 112 ± 12 | 98 ± 9 |  | 82 ± 8 | 77 ± 6 |
| Alcohol (g) | 7.82 ± 1.86 | 4.70 ± 1.41 |  | 7.23 ± 1.58 | 4.97 ± 1.42 |
| Cholesterol (mg) | 407 ± 53 | 355 ± 38 |  | 314 ± 36 | 312 ± 31 |
| Total Saturated Fatty Acids (g) | 35.7 ± 4.3 | 32.9 ± 3.9 |  | 27.4 ± 2.9 | 24.9 ± 2.7 |
| Total Sugars (g) | 125 ± 13 | 119 ± 14 |  | 98 ± 9 | 90 ± 9 |
| Dietary fiber (g) | 22.5 ± 2.1 | 21.8 ± 1.9 |  | 17.5 ± 1.7 | 15.8 ± 1.0 |
| Vitamin A (mcg) | 1245 ± 159 | 9601 ± 90 |  | 875 ± 122 | 771 ± 79 |
| Vitamin E (mg) | 11.3 ± 1.1 | 10.2 ± 0.9 |  | 9.1 ± 1.0 | 7.6 ± 0.6 |
| Vitamin K (mcg) | 393 ± 101 | 220 ± 36 |  | 264 ± 74 | 174 ± 26 |
| Vitamin C (mg) | 134 ± 18 | 124 ± 16 |  | 96 ± 13 | 78 ± 6 |
| Vitamin B1 (mg) | 2.02 ± 0.19 | 1.82 ± 0.17 |  | 1.59 ± 0.17 | 1.42 ± 0.11 |
| Vitamin B2 (mg) | 3.25 ± 0.35 | 2.88 ± 0.29 |  | 2.56 ± 0.26 | 2.37 ± 0.24 |
| Niacin (mg) | 29.7 ± 3.0 | 27.4 ± 2.8 |  | 24.0 ± 2.5 | 20.8 ± 1.7 |
| Vitamin B6 (mg) | 2.62 ± 0.27 | 2.32 ± 0.24 |  | 2.05 ± 0.22 | 1.74 ± 0.14 |
| Folate (mcg) | 568 ± 52 | 518 ± 53 |  | 450 ± 49 | 399 ± 28 |
| Vitamin B12 (mcg) | 7.60 ± 1.00 | 6.92 ± 0.78 |  | 5.86 ± 0.90 | 5.54 ± 0.59 |
| Calcium (mg) | 1411 ± 177 | 1268 ± 124 |  | 1029 ± 111 | 987 ± 95 |
| Phosphorous (mg) | 1866 ± 201 | 1677 ± 151 |  | 1361 ± 132 | 1308 ± 105 |
| Magnesium (mg) | 442 ± 39 | 396 ± 30 |  | 339 ± 29 | 297 ± 19 |
| Iron (mg) | 19.0 ± 1.8 | 17.4 ± 1.7 |  | 15.3 ± 1.8 | 13.8 ± 1.0 |
| Zinc (mg) | 16.6 ± 1.7 | 14.9 ± 1.5 |  | 12.8 ± 1.5 | 11.6 ± 1.0 |
| Copper (mg) | 1.88 ± 0.16 | 1.75 ± 0.13 |  | 1.49 ± 0.13 | 1.28 ± 0.07 |
| Selenium (mcg) | 146 ± 16 | 135 ± 12 |  | 113 ± 11 | 106 ± 8 |
| Sodium (mg) | 4124 ± 377 | 3895 ± 380 |  | 3231 ± 295 | 3033 ± 227 |
| Potassium (mg) | 4144 ± 380 | 3659 ± 301 |  | 3116 ± 299 | 2851 ± 216 |

Values are means ± SEM

There were no significant differences between placebo and CEB at wk 0 and wk 8, analyzed by ANOVA using the test parameter estimate for the main effect of treatment in our model (P <0.05)

**Supplemental Table 3** Concentrations (ng/mg creatinine) of flavanols, flavonols, and phenolic acids in fasting morning spot urine samples collected following 8 wk daily consumption in participants assigned to consumed placebo (*n* = 38) or CEB (*n* = 40)

|  |  | Placebo | CEB |
| --- | --- | --- | --- |
| Epicatechin |  | 70 ± 23 | 74 ± 24 |
| Quercetin |  | 138 ± 26 | 176 ± 20 |
| Caffeic acid |  | 18 ± 4 | 19 ± 4 |
| p-Coumaric acid |  | 27 ± 8 | 44 ± 11 |
| Ferulic acid |  | 310 ± 57 | 217 ± 29 |
| Sinapic acid |  | 27 ± 7 | 18 ± 3 |
| 4-OH-PAA |  | 11304 ± 1360 | 9542 ± 1217 |
| 3,4-OH-PAA |  | 380 ± 28 | 374 ± 27 |
| 4-OH-3-MeOH-PAA |  | 4138 ± 252 | 4332 ± 279 |
| 3-OH-Benzoic acid |  | 2489 ± 347 | 2950 ± 429 |
| Gentisic acid |  | 140 ± 28 | 133 ± 22 |

Values are means ± SEM

There were no significant differences between placebo and CEB at wk 8, analyzed by ANCOVA, with baseline values as a covariate, using the test parameter estimate for the main effect of treatment in our model (P <0.05)

**Supplemental Table 4** Concentrations (ng/mg creatinine) of flavanols, flavonols, and phenolic acids in pooled 24 h urine samples collected at baseline (wk 0) and following 8 wk daily consumption of CEB (*n* = 40)

|  | Wk 0 | Wk 8 |
| --- | --- | --- |
| Epicatechin | 28 ± 10 | 140 ± 35* |
| Quercetin | 340 ± 25 | 365 ± 25 |
| Caffeic acid | 36 ± 11 | 81 ± 17* |
| p-Coumaric acid | 43 ± 8 | 159 ± 21* |
| Ferulic acid | 539 ± 43 | 1020 ± 119* |
| Sinapic acid | 126 ± 24 | 278 ± 38* |
| 4-OH-PAA | 8864 ± 482 | 8689 ± 433 |
| 3,4-OH-PAA | 532 ± 25 | 618 ± 30* |
| 4-OH-3-MeOH-PAA | 3503 ± 127 | 4026 ± 128* |
| 3-OH-Benzoic acid | 2566 ± 270 | 2456 ± 257 |
| Gentisic acid | 195 ± 20 | 294 ± 31* |

Values are means ± SEM

Flavanols, flavonols, and phenolic acids were not measured in 24-h pooled urine samples collected from the placebo group

Symbols denote significant difference within treatment compared to baseline (wk 0) values, analyzed by ANOVA using the test parameter estimate for the main effect of time in our model (**P* <0.05)

**References**

1. United States Pharmacopeial Convention (2010) Food Chemicals Codex 7th ed. United Book Press, Inc., Baltimore
2. Martin MA, Ramos S, Mateos R, Marais JP, Bravo-Clemente L, Khoo C, Goya L (2015) Chemical characterization and chemo-protective activity of cranberry phenolic powders in a model cell culture. Response of the antioxidant defenses and regulation of signaling pathways. Food Rese Int 71:68-82
3. Prior RL, Fan E, Ji H, Howell A, Nio C, Payne MJ, Reed J (2010) Multi-laboratory validation of a standard method for quantifying proanthocyanidins in cranberry powders. J Sci Food Agric 90:1473-1478
4. Chen CY, Milbury PE, Collins FW, Blumberg JB (2007) Avenanthramides are bioavailable and have antioxidant activity in humans after acute consumption of an enriched mixture from oats. J Nutr 137:1375-1382
5. Chen CY, Milbury PE, Lapsley K, Blumberg JB (2005) Flavonoids from almond skins are bioavailable and act synergistically with vitamins C and E to enhance hamster and human LDL resistance to oxidation. J Nutr 135:1366-1373
